# Supplementary material for: Hybrid Models and Biological Model Reduction with PyDSTool
Source: PLoS Comput Biol. 2012 Aug 9;8(8):e1002628. doi: 10.1371/journal.pcbi.1002628 (PMC3415397; doi:10.1371/journal.pcbi.1002628)
Supplement: Text S4 — Complete source code for the PyDSTool package (version 0.88.120504). Includes API documentation and help files linking to web pages. This file is identical to the current public release on Sourceforge.net. (ZIP) [file pcbi.1002628.s004.zip › PyDSTool/html/PyDSTool.Toolbox.dataanalysis.data_bins-class.html]

xml version="1.0" encoding="ascii"?


PyDSTool.Toolbox.dataanalysis.data\_bins


| Home | Trees | Indices | Help | PyDSTool |
| --- | --- | --- | --- | --- |

Package PyDSTool ::
Package Toolbox ::
Module dataanalysis ::
Class data\_bins

[hide private]

[frames] | no frames]

# Class data\_bins

source code

```
object --+
         |
        data_bins
```

---

```
Class for data binning. Indexed by bin number 0 - (num_bins-1).

Initialization arguments:
        coordinate -- name of coordinate that occupies bins
        bin_ords   -- ordered sequence of n bin ordinates (integers or floats)
                      defining n-1 bins
        valuedict  -- optional dictionary containing initial values for
                      bins: keys = ordinate values,
                            values = coordinate values
        tolerance  -- for resolving bin edges with finite precision
                      arithmetic (defaults to 1e-12)
```


|  |  |
| --- | --- |
| Instance Methods | [hide private] |

|  |  |
| --- | --- |
| \_\_init\_\_(self, coordinate, bin\_ords, valuedict=None, tolerance=1e-12)  x.\_\_init\_\_(...) initializes x; see x.\_\_class\_\_.\_\_doc\_\_ for signature | source code |

|  |  |
| --- | --- |
| resolve\_bin\_index(self, ordinate)  Find bin number (index) associated with ordinate | source code |

|  |  |
| --- | --- |
| \_\_call\_\_(self, ordinate)  Return content of the bin that ordinate resolves to. | source code |

|  |  |
| --- | --- |
| \_\_getitem\_\_(self, bin\_index) | source code |

|  |  |
| --- | --- |
| \_\_setitem\_\_(self, bin\_index, value)  Set value of a given bin number (index). | source code |

|  |  |
| --- | --- |
| increment(self, ordinate, inc=1)  Increment the bin corresponding to the ordinate argument. | source code |

|  |  |
| --- | --- |
| bin(self, values)  Increment bin contents according to values passed. | source code |

|  |  |
| --- | --- |
| clear(self)  Reset all bins to have zero contents. | source code |

|  |  |
| --- | --- |
| \_\_str\_\_(self)  str(x) | source code |

|  |  |
| --- | --- |
| \_\_repr\_\_(self)  str(x) | source code |

|  |  |
| --- | --- |
| to\_pointset(self)  Convert to a pointset | source code |

|  |  |
| --- | --- |
| to\_arrays(self)  Convert to two arrays of bin indices and values | source code |

|  |  |
| --- | --- |
| values(self) | source code |

|  |  |
| --- | --- |
| keys(self) | source code |

|  |  |
| --- | --- |
| mean(self)  Mean of binned data | source code |

|  |  |
| --- | --- |
| std(self)  Standard deviation of binned data | source code |

**Inherited from `object`**:
`__delattr__`,
`__getattribute__`,
`__hash__`,
`__new__`,
`__reduce__`,
`__reduce_ex__`,
`__setattr__`


|  |  |
| --- | --- |
| Properties | [hide private] |

**Inherited from `object`**:
`__class__`


|  |  |
| --- | --- |
| Method Details | [hide private] |

|  |  |
| --- | --- |
| \_\_init\_\_(self, coordinate, bin\_ords, valuedict=None, tolerance=1e-12)  *(Constructor)* | source code |

x.\_\_init\_\_(...) initializes x; see x.\_\_class\_\_.\_\_doc\_\_ for
signature

Overrides: object.\_\_init\_\_: *(inherited documentation)*

|  |  |
| --- | --- |
| increment(self, ordinate, inc=1) | source code |

Increment the bin corresponding to the ordinate argument. Optional
argument inc sets increment amount (default 1).

|  |  |
| --- | --- |
| \_\_str\_\_(self)  *(Informal representation operator)* | source code |

str(x)

Overrides: object.\_\_str\_\_: *(inherited documentation)*

|  |  |
| --- | --- |
| \_\_repr\_\_(self)  *(Representation operator)* | source code |

str(x)

Overrides: object.\_\_repr\_\_: *(inherited documentation)*

  


| Home | Trees | Indices | Help | PyDSTool |
| --- | --- | --- | --- | --- |

|  |  |
| --- | --- |
| Generated by Epydoc 3.0.1 on Fri May 4 15:24:08 2012 | http://epydoc.sourceforge.net |
